# Metabolism and metabolomics of ketamine: a toxicological approach

**DOI:** 10.1080/20961790.2017.1285219

**Published:** 2017-02-20

**Authors:** Ricardo Jorge Dinis-Oliveira

**Affiliations:** a Department of Sciences, IINFACTS – Institute of Research and Advanced Training in Health Sciences and Technologies, University Institute of Health Sciences (IUCS), CESPU, CRL, Gandra, Portugal; b Department of Biological Sciences, UCIBIO, REQUIMTE, Laboratory of Toxicology, Faculty of Pharmacy, University of Porto, Porto, Portugal; c Department of Public Health and Forensic Sciences, and Medical Education, Faculty of Medicine, University of Porto, Porto, Portugal

**Keywords:** Forensic science, forensic toxicology, ketamine, metabolism, metabolomics, toxicity

## Abstract

Ketamine is a phencyclidine derivative and a non-competitive antagonist of *N*-methyl-*D*-aspartate (NMDA) receptor for which glutamate is the full agonist. It produces a functional dissociation between the thalamocortical and limbic systems, a state that has been termed as dissociative anaesthesia. Considerable variability in the pharmacokinetics and pharmacodynamics between individuals that can affect dose-response and toxicological profile has been reported. This review aims to discuss pharmacokinetics of ketamine, namely focusing on all major and minor, active and inactive metabolites. Both ketamine optical isomers undergo hepatic biotransformation through the cytochrome P450, specially involving the isoenzymes 3A4 and 2B6. It is first *N-*demethylated to active metabolite norketamine. Different minor pathways have been described, namely hydroxylation of the cyclohexanone ring of ketamine and norketamine, and further conjugation with glucuronic acid to increase renal excretion. More recently, metabolomics data evidenced the alteration of several biological pathways after ketamine administration such as glycolysis, tricarboxylic acid cycle, amino acids metabolism and mitochondrial β-oxidation of fatty acids.

It is expected that knowing the metabolism and metabolomics of ketamine may provide further insights aiming to better characterize ketamine from a clinical and forensic perspective.

## Introduction

Ketamine is a synthetic, non-barbiturate, injectable dissociative anaesthetic first synthesized by Calvin Stevens of the Parke-Davis Pharmaceutical Company in 1962 (Ann Arbor, Michigan) while searching for an alternative to the potent hallucinogenic agent phencyclidine [1,2]. Due to its quick onset and short duration of action with only slight cardio-respiratory depression in comparison with other general anaesthetics and the possibility of inhalation to maintain the anaesthetic state, ketamine is a preferred drug for short-term surgical procedures in veterinary and human medicine, especially in children [3,4]. Indeed, in adults, it induces severe psychomimetic reactions, namely hallucinations, delirium, nightmares, altered of short-term memory and cognition [5–7]. It has also been proposed as analgesic and for the treatment of alcoholism [8], heroin addiction [9], anorexia [10] and for the treatment of depression due to its long-lasting effects and rapid onset of action (within 4 h post-administration) [11–14].

Ketamine produces dissociative anaesthesia (i.e. sense of dissociation from the body and the environment), a neologism first coined by Corssen and Domino [15,16]. This means that the patient remains conscious and appears to be awake (i.e. eyes may be open with presence of nystagmus) but exhibit no apparent response to surgical pain; “the lights are on, but no one's home” [17]. Represents a “trancelike cataleptic state” characterized by profound and complete analgesia and total amnesia with preservation of protective airway reflexes (i.e. intubation is unnecessary), spontaneous respirations and cardiovascular stability (i.e. blood pressure and pulse rate do not decrease and may even increase slightly) [6,17]. The dissociative state seems to result from a functional dissociation: inhibition of thalamocortical pathways and stimulation of the limbic regions of the brain [18]. These neuronal systems help to maintain neuronal connections required for consciousness.

Ketamine is primarily a stereoselective non-competitive antagonist of the ionotropic receptor of *N*-methyl-*D*-aspartate (NMDA) reducing calcium ion influx through this channel and therefore prevents neuronal activation required for conscious state [19,20]. This effect at NMDA receptors is able to reverse the enhanced pain sensitivity that is frequently present in major trauma or surgical injury and increases the antinociceptive effects of conventional opioid and nonsteroidal anti-inflammatory drugs (NSAIDs) [21,22]. In addition, ketamine also exerts non-NMDA related effects, interacting with several receptors, namely [19,23–27]: (1) μ, κ and δ opioid receptors, that contributes to its analgesic effects; (2) anticytokine effect; (3) inhibition of acetylcholine muscarinic and nicotinic receptors; (4) inhibition of L-type calcium and sodium channels current; (5) adrenergic receptors; (6) serotonin receptors; (7) dopaminergic D_2_ receptors; and (8) inhibition of neuronal sodium channels (producing a modest local anaesthetic action). In opposition to several other anaesthetics, it does not affect γ-aminobutyric acid receptors at clinically relevant concentrations [28].

**Figure 1.**
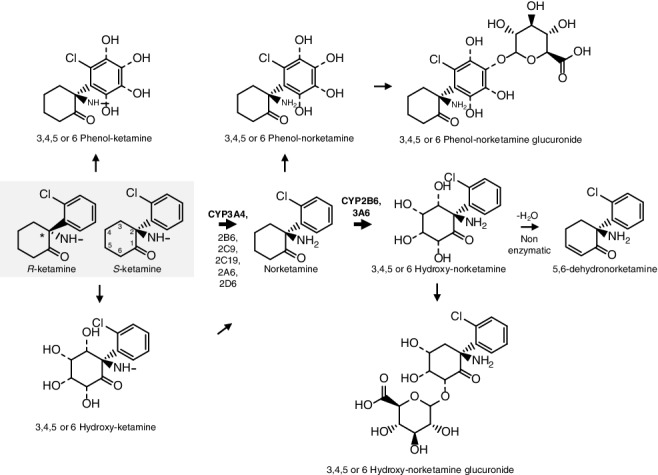
Metabolism of ketamine.

As shown in Figure 1, ketamine [2-(O-chlorophenyl)-2-(methylamino)-cyclohexanone] has one stereogenic centre in the C2 position of the molecule and therefore can exist as two possible stereoisomers [*R* (*l*-ketamine) and *S* (*d*-ketamine; esketamine)]. Differences in pharmacological effects and pharmacokinetic properties between the two enantiomers have been described *in vivo* and *in vitro* [29,30]. The affinity of *S*-ketamine was demonstrated to be four times higher for the phencyclidine site of the NMDA receptor when compared with *R*-ketamine [31,32], resulting in a likewise increase in the hypnotic/anesthetic properties of the *S*-enantiomer [29] and its analgesic potential was reported to be twice that of the racemic mixture and four times that of *R*-ketamine [33]. Originally, it was used as racemic mixture, but nowadays in human medicine, *S*-ketamine is preferable due to its higher potency together with faster post-anaesthetic recovery times [3,4]. Unfortunately, the drug is still used as the racemic mixture.

One of the objectives of metabolism and metabolomics is related to the qualitative and quantitative characterization of all pictures of biochemical and biological metabolic processes of an organism (i.e. the metabolome) and their changes over time [34]. Several studies have demonstrated an unpredictable inter-individual variability of ketamine pharmacokinetics and pharmacodynamics [35,36]. Moreover, metabolic substrates and/or inhibitors or inducers of the same cytochrome P450 isoforms (CYP) implicated in ketamine metabolism are administered concurrently and thus important clinical and forensic consequences are expected. This work aims to perform a literature review of ketamine biotransformation and metabolomics, their pharmacological, toxicological effects, which have not been characterized sufficiently in most studies.

## Methodology

An English exhaustive literature search was carried out to identify relevant articles. Ketamine metabolizing enzymes and metabolites, and metabolomics were searched in PubMed (U.S. National Library of Medicine) without a limiting period. Furthermore, electronic copies of the full papers were obtained from the retrieved journal articles as well as books on ketamine and then further reviewed to find possible additional publications related to human and non-human, *in vivo* and *in vitro* studies.

## Absorption, distribution and excretion

Since it is water- and lipid-soluble xenobiotic, ketamine can be administered via almost any route depending on the intent. Oral, inhaled, rectal, smoked, intramuscular, subcutaneous, intravenous, epidural and intrathecal are the most frequently applied routes [28,32,37]. Oral ketamine administration undergoes significant first-pass effects leading to the formation of norketamine and dehydronorketamine (Figure 1) [38]. Rectal administration has a more rapid onset of action and has been applied specially in children [39]. When used as a recreational drug, ketamine is usually inhaled (insufflated). Usually, the liquid commercial legal anaesthetic form is allowed to evaporate before administration [32]. Because of ketamine crystalline appearance, the drug can be mistaken for or passed off as methamphetamine and cocaine. Bioavailabity largely depends on the route of administration (e.g. 20% oral; 90% intramuscular; 25% rectal; 50% intranasal; 77% epidural) [32,40,41].

Since ketamine is very lipid-soluble and has a relatively low protein binding (about 20%–50%), a very large volume of distribution (3–5 L/kg) is attained [32,42]. Therefore, it rapidly crosses the blood–brain barrier to induce anaesthesia although the onset time is slower than thiopental. The affinity for α_1_-acid glycoprotein seems to be much higher than albumin [43]. After intramuscular or subcutaneous injection, sedation or anaesthesia develops within 10–15 minutes and typically lasts for 30–120 minutes [5]. When administered intravenously, the onset of action typically occurs within 1–2 minutes and anaesthesia lasts for approximately 20–60 minutes [5]. After oral administration, the action onset typically occurs within 20–30 minutes and the duration of effect is between 60 and 90 minutes [5]. Termination of anaesthesia is due to redistribution from the brain and plasma to other tissue. Ketamine is a week base with a pKa of 7.5 [44]. At physiological pH of 7.4, it is 44.3% un-ionized [45]. Since its pKa is close to physiological pH, small changes in pH result in a wide variation in the ionized and non-ionized fractions.

Analogous to other anaesthetics such as barbiturates and propofol, the elimination half-life is short; approximately 2–4 h by intravenous route [46]. The pharmacokinetics in children is not very different from adults, although children do form more norketamine than adults [47]. Indeed, children require higher infusion rates than adults to maintain the ketamine steady-state probably attributed to age-related pharmacokinetics [48].

Ketamine and metabolites are excreted in urine; 2% is excreted unchanged, 2% in the form of norketamine, 16% as dehydronorketamine and 80% as conjugates of hydroxylated ketamine metabolites with glucuronic acid [32,49]. Other studies on human subjects given tritium-labelled ketamine intravenously have shown that while 91% of the administered radioactivity could be recovered in urine over a period of five days [50], only 20% of the dose is presented as parent drug, norketamine and 5,6-dehydronorketamine [51]. This means that a great proportion of an intravenous dose of ketamine is converted to other metabolites whose chemical structure and pharmacological activity are yet to be established. Hydroxylated metabolites of the parent drug and/or norketamine may be formed *invivo* and subsequently eliminated in urine and bile as conjugates [20,52]. Several studies described that frequently repeated doses of ketamine prolonged its elimination time as long as 11 days [49,53]. In another study, norketamine was detected in urine samples up to 14 days after administration of a single intravenous dose of ketamine to children [49].

## Metabolism

The metabolism of ketamine has been described as extensive and stereoselective and occurs mainly in the liver [52]. The major metabolic pathway is *N*-demethylation to an active metabolite norketamine by CYP3A4 (Figure 1) [18]. Norketamine, besides some psychoactive properties, also retains anaesthetic effects; this fact explains the maintenance of the therapeutic efficacy at lower blood ketamine concentrations [32]. In humans, at therapeutic concentrations, CYP2B6, CYP3A4 and CYP2C9 make only a minor contribution to ketamine *N*-demethylation [54]. Nevertheless, there are some dissonant results regarding enzymes comparative contributions to clinical ketamine metabolism [55]. Non-human data indicate that norketamine crosses the blood–brain barrier and has about one-fifth to one-third the potency of ketamine, contributing to the analgesic and psychomimetic side effects, especially in long infusions or chronic use [56–58]. Paul and colleagues [26] demonstrated that besides ketamine, also norketamine and hydroxynorketamine increase the levels of the activating phosphorylated form of mammalian target of rapamycine (mTOR; a protein kinase involved in protein synthesis, synaptic plasticity and neurotrophic signalling) and cognate signalling kinases *in vitro* and prefrontal cortex in the rat.

Ketamine and norketamine are also hydroxylated at 3–6 carbons of the cyclohexanone ring leading to the formation of both inactive free and glucuronidated hydroxylated derivatives, which are more water-soluble compounds in order to facilitate urinary excretion [55,59–62].

The hydroxylation of the alicyclic ring led to the introduction of a second chiral centre and thereby diastereomers with two chiral centres are formed. Although previous studies did not show formation of phenolic metabolites of ketamine [63], more recent studies evidence their existence in urine of subjects after oral ketamine administration [62].

Cyclohexanone ring also undergoes oxidative metabolism by dehydrogenation to an active metabolite 5,6-dehydronorketamine [64]. Only the 5-hydroxynorketamine stereoisomers proved to be precursors of 5,6-dehydronorketamine [60,65,66]. 5,6-dehydronorketamine has been posted as the most useful metabolite to target in a forensic context since it has a longer plasma half-life (i.e. detected for 6–10 days in volunteers) [3,67]. Hydroxylated dehydronorketamine or dihydroxynorketamine metabolites were not obtained from microsomal incubates of ketamine or norketamine [62].

## Drug–drug interactions and pharmacogenomics in metabolism

Since ketamine undergoes extensive metabolism, drug–drug interactions and inter-individual variability due to polymorphisms in genes coding for drug-metabolizing enzymes are expected to result in clinically important consequences. However, there are few reports about drug interactions and pharmacogenomics of ketamine in humans. The treatment with diazepam, a substrate of CYP2C19 and CYP3A4, or secobarbital, an inhibitor of CYP2B, increased plasma half-lives of ketamine in humans and therefore their sedative effects [68,69]. Since ketamine decreases CYP3A enzyme activity, diazepam metabolism may be also slowed [70]. When ketamine was associated with bupivacaine, local anaesthetic effect was significantly enhanced as well as its elimination half-life [71]. In rats, ketamine also decreases the clearance of the CYP2D1 substrate flecainide and the CYP3A substrate ethosuximide, by 13% and 18%, respectively [72]. Pre-treatment of rat and rabbit with phenobarbital, an inducer of the CYP2B subfamily, causes a marked increase in the rate of ketamine metabolism by hepatic tissue *in vitro* [65]. Rifampicin, a potent inducer of many CYP enzymes, particularly of CYP3A, significantly reduces the plasma concentrations of ketamine and norketamine after oral administration of *S*-ketamine; this effect was only moderately registered after its intravenous administration [73,74]. Clarithromycin, a potent inhibitor of CYP3A and P-glycoprotein transporter, also increased serum *S*-ketamine concentrations (263%) [75]. Unexpectedly, itraconazole, a potent inhibitor of CYP3A and P-glycoprotein transporter, had no effect on the pharmacokinetics of *S*-ketamine, whereas inhibition of CYP2B6 by ticlopidine significantly increased its serum concentrations, emphasizing the role of CYP2B6 in the elimination of ketamine [74]. Further studies are needed to evaluate the influence of polymorphisms of CYP3A4, but especially for CYP2B6 and/or CYP2C9, on ketamine pharmacokinetics. Until now, a significant impact of the CYP2B6*6 allele (i.e. the most prevalent and clinically important variant) on *N*-demethylation of ketamine was registered *in vitro* [76]. Corroborating these results, CYP2B6*6 allele was associated with reduced steady-state ketamine plasma clearance in chronic pain patients [36]. Authors hypostatized that the higher plasma concentrations obtained may predispose to higher incidence of ketamine adverse effects.

Ketamine also causes “self-induction” of multiple hepatic P450 isoforms in rat liver microsomes (i.e. 1A, 2B, 2E1 and 3A proteins by 2-, 13-, 2- and 2-fold, respectively), meaning that after chronic administration of ketamine, higher doses may be required to obtain the therapeutic effect [77,78]. There is some evidence that cocaine may interact with ketamine to cause hepatic injury, at least in experimental animals [77]. The effect strongly suggests that due to ketamine P450 induction, liver toxicity may increase after exposure to cocaine and/or increase formation of norcocaine (i.e. the main hepatotoxic oxidative metabolite) [79]. Nevertheless, results are not conclusive since other authors have shown that gene expression of CYP3A4 was suppressed by ketamine by disturbing cytoskeleton remodelling due to reduction of calcium mobilization and adenosine triphosphate (ATP) synthesis [80]. Perhaps, differences in experimental designs may explain such discrepancies since ketamine need time to achieve its inductive effect [81]. Indeed, while single-dose administration of ketamine inhibited CYP3A4-involved *N*-demethylation of erythromycin [80], repetitive exposure can induce CYP activities, similarly to ethanol [82,83].

Moreover, some studies demonstrated that ketamine noncompetitively inhibits the glucuronidation of morphine to morphine-3-glucuronide in rats [38]. This result may explain the prolonged duration of morphine-induced analgesia, less administrations and reduced adverse effects of morphine in humans [15,16]. Since ketamine is often used in combination with other drugs, especially cocaine and heroin (for which morphine is the active metabolite), these possible interactions should not be disregarded [79,84,85]. Insights are needed to explain mechanisms of alterations of CYP activities since these may compromise drug metabolism, drug–drug interactions and the toxicity and carcinogenicity of foreign chemicals [81].

## Endogenous metabolome

Besides exposure metabolites, several other endogenous metabolic changes are expected after ketamine administration, but were not yet well explored in detail. Zhang and colleagues [86] provide data regarding serum metabolites changes due to ketamine abuse in rats. Authors shown that ketamine induced alterations in the levels of phosphate, propanoic acid, butanoic acid, ribitol, propanetricarboxylic acid, hexadecanoic acid, *d*-fructose, *l*-leucine, *l*-threonine, alanine, glycine, butanoic acid, valine, *l*-serine, *l*-proline, mannonic acid, octadecanoic acid and cholesterol. Particularly important are the phosphate, propanoic acid, ribitol and *d*-fructose alterations, which are intermediate substances in energy metabolism. Propanoic acid is a product of glycolysis that can be metabolized into propionyl-CoA and then enters the tricarboxylic acid cycle (TCA cycle). Moreover, since phosphate is used to ATP synthesis, ketamine abuse in rats may result in changes in the energetic status of cells. Regarding alanine, glycine, butanoic acid, valine, *l*-serine, *l*-proline, malfunctioned protein synthesis and degradation are expected. Ketamine may be also implicated in liver damage since lipid metabolism was also compromised. In other study, Wen et al. [87] observed that compared with the control group, the levels of glycerol, uridine and cholesterol in rat brain of the ketamine group decreased, while the urea levels increased. Glycerol and fatty acids are released into blood when the body uses stored fat as a source of energy. Uridine plays a role in the glycolysis pathway of galactose, which is converted to glucose and metabolized in the common glucose pathway. Urea is an end product of the metabolism of nitrogen containing compounds by animals that is excreted in urine. Authors also found biomarkers differently expressed in plasma and brain: compared with the control group, the levels of butanoic acid, phosphate, aminomalonic acid, gluconic acid, hexadecanoic acid, oleic acid and arachidonic acid increased in the plasma of the ketamine group, while the levels of glycine, *l*-lysine and cholesterol decreased [87]. Several hippocampal pathways including glycolysis/gluconeogenesis, pentose phosphate pathway and citrate cycle are also affected after ketamine administration [88].

An interesting study was performed by Villaseñor et al. [89]. Authors performed a metabolomic analysis of plasma samples from patients receiving ketamine for the treatment of bipolar depression. Results evidenced that lysophosphatidylethanolamines and lysophosphatidylcholines increased in responder's patients suffering from resistant bipolar depression. These findings suggest that alterations in the mitochondrial β-oxidation of fatty acids are not registered in ketamine-administered group. Authors concluded that metabolomics may be useful to predict response aiming a ketamine personalized therapy. More recently, Rotroff and colleagues [90] used metabolomics to provide new insights aiming to map global blood metabolic effects of ketamine racemic mixture and *S*-ketamine in treatment-refractory major depressive disorder patients. Tryptophan, the amino-acid precursor of serotonin, and related metabolites such as indole-3-acetate, indole-3-lactate and tyrosine, the amino-acid precursor of dopamine, were all decreased after ketamine administration. However, neither dopamine nor serotonin levels were significantly changed, meaning that further studies are required. Authors also observed that glutamate and circulating phospholipids (e.g. phosphatidylcholine and phosphoethanolamine) levels were significantly increased and were associated with decreases in depression severity. Since ketamine blocks the glutamatergic NMDA receptor, thus the possible effect of increased glutamate levels could shift glutamatergic signalling from NMDA to α-amino-3-hydroxy-5-methyl-4-isoxazolepropionic acid receptor receptors to enhance the 5HT_1B_ receptor activity that is claimed to be required for antidepressant effects [91]. Moreover, since phosphatidylcholine is a major component of cell membranes, authors hypothesized that their increased synthesis is greater in the patients whose depression severity is decreasing. Other structurally non-identified metabolites also proved to be potentially interesting.

Pan and colleagues [92] demonstrated that ketamine influenced the major energy and amino acid metabolic pathways in macaques. Indeed, serum levels of α-glucose, myoinositol, lactate and succinate, and urine levels of pyruvate and lactate were all decreased. In contrast, the levels of leucine in serum and arginine in urine were significantly higher in the ketamine group.

## Conclusion and future perspectives

For more than 50 years, ketamine has been widely used to induce anaesthesia and to produce analgesia [6]. Nevertheless, its use is limited due to its unfavourable psychoactive effects. In contrast to classic hallucinogens (e.g. lysergic acid diethylamide, psilocybin, mescaline, etc.), ketamine presents a higher risk of dependence and addiction (i.e. to develop a compulsive drug-seeking behaviour) [1]. Indeed, hallucinogens (i.e. compounds able to change in lower doses the perception of reality regarding thought, time and space and alterations of humour and consciousness, without causing marked psychomotor stimulation or depression) differ from most other psychoactive drugs since they induce neither dependence nor addiction nor are used for prolonged periods; in other words, these drugs do not interfere with mesolimbic rewarding system [66]. However, repetitive exposure causes rapid tolerance (also called tachyphylaxis) leading abusers to escalate doses to achieve the full hallucinogenic experience [66,93]. In recent years, ketamine has been used as a recreational and “club drug” due to its hallucinogenic, dream-like state, sensation of floating outside the body, “cosmic” experiences, body distortions and stimulant effects at dance parties and raves [94–96]. After being illegally diverted from legal suppliers (e.g. veterinary), ketamine has also been misused as a “date-rape” drug since it induces amnesia as γ-hydroxybutyric acid and flunitrazepam [97]. The narcotic effects of ketamine are comparable to those achieved by phencyclidine, and the hallucinogen action is similar to that of lysergic acid diethylamide [59].

Although ketamine therapy is generally considered safe, several toxicological consequences can be highlighted [49]: (1) at lower doses it causes mild intoxication, dreamy thinking, alterations of speech, hearing and seeing, muscular discoordination, disorientation, anxiety, disinhibition, euphoria, seeing the world differently and irrational behaviour; (2) higher doses cause great difficulty in moving, respiratory disturbances, seizures and nausea; (3) extreme doses produce complete dissociation from reality and loss of consciousness, hallucinations, out-of-body experiences and so-called “near-death experiences” or the “K-hole” [93]; (4) in absence of other drugs, deaths by overdose (e.g. due to respiratory depression) are rare since doses used by addicts are typically lower than necessary for therapeutic anaesthesia. Accidental deaths have been reported as consequence of falling, hypothermia, traffic accident or drowning [75]; and (5) the frequent use of ketamine can lead to addiction and dependence [98]. Sometimes ketamine abused is mixed with other drugs such as cocaine, methamphetamine, 3,4-methylenedioxymethamphetamine and benzodiazepines [99].

Despite some studies, the metabolism and metabolomics of ketamine remain poorly understood. In this work, exogenous and endogenous metabolome of ketamine was fully reviewed. Ketamine undergoes hepatic *N*-demethylation by several isoforms of cytochrome P450 to norketamine, which is the main metabolite excreted in urine. In order to better comprehend clinical effects, pharmacokinetic studies should focus on both active and inactive metabolites and on polymorphisms in genes encoding enzymes (e.g. CYP2B6) involved in ketamine metabolism [100,101]. Moreover, due to its extensive metabolism, potentially dangerous interactions are expected when other drugs are taken together. The identification of additional metabolites will be particularly useful to confirm xenobiotic exposure [102] and further sensitive analytical methods are needed to prove consumption in a wider detection window [49]. Since ketamine has become a popular club and date-rape drug, research on biotransformation acquires particular importance in forensic toxicology. Moreover, the mechanisms underlying the emergence psychedelic alterations are not fully understood. Based on the time course of their appearance and due to the low levels of ketamine registered, active metabolites are expected to be involved. Scarce data exist in the literature regarding the binding of ketamine and metabolites such as norketamine and dehydronorketamine to serum proteins namely to albumin and α_1_-acid glycoprotein. The clinical relevance for patients with serum protein levels severely altered also needs to be further explored. Finally, particularly interesting will be the metabolomics elucidation of the mechanisms by which ketamine rapidly treats depressive symptoms. To be accomplished, the identification of unknown metabolites may provide novel insights into the biological pathways involved.
